# Gaucher's disease: report of 11 cases with review of literature

**DOI:** 10.11604/pamj.2015.20.18.4112

**Published:** 2015-01-07

**Authors:** Laila Essabar, Toufik Meskini, Najat Lamalmi, Said Ettair, Naima Erreimi, Nezha Mouane

**Affiliations:** 1 FMPR, University Mohammed V Souissi, Department of Pediatric Hepatology Gastroenterology and Nutrition – P III, Rabat Children's Hospital, Morocco; 2 FMPR, University Mohammed V Souissi, Department of Anatomo-Pathology, Rabat Children's Hospital

**Keywords:** Gaucher Disease, children, hepatosplenomegaly, enzymatic dosage, splenectomy

## Abstract

Gaucher's disease (GD) is a lysosomal storage disorder due to glucocerebrosidase deficiency; it's one of the rare genetic diseases for which therapy is now available. The purpose of this work is to study the epidemiological features of the disease and to highlight the diagnostic difficulties. We performed an 11-year retrospective study of 11 patients with GD followed-up in the department of paediatric hepatology gastroenterology and nutrition of Rabat children's Hospital. We observed 11 patients with GD: 6 males and 5 females. Age at onset ranged from 3 months to 10 years with an average of 3.41 years. Mean age at diagnosis was 4 years (range 3months-14years). Parental consanguinity was noted in 85% cases. According to the clinical presentation, we classified our patients into: 9 cases of type 1 (81%) and two cases of type 2 (19%), none of the patients presented GD type 3. GD type 1: The age at diagnosis ranged from 2 years to 14 year with an average of 6 years. Main symptoms were: splenomegaly, hepatomegaly, pallor, haemorrhagic appearance (40%), bone pain (40%). The diagnosis was based on histology showing the Gaucher's cells in various tissues (100%). Enzymatic activity dosage confirmed the diagnosis of GD for 4 patients (44.5%). The treatment was always symptomatic (analgesics, transfusion). A splenectomy was performed in one case presenting with multiple splenic abscesses and high transfusion requirements. None of the patients received a specific treatment (substitutive enzymotherapy). The follow-up period ranged from 3 months to 6 years with an average follow-up of 4 years. We noticed stability in 4 cases, 2 worsening cases with bone and spleen complications. Three patients were lost to follow-up. GD type 2: we observed two cases of GD type 2 diagnosed at 3 and 18 months. The visceral symptoms were serious and the neurological features included seizures, hypertony, squint, physical developmental milestones delay. Both of them died. Gaucher's disease is not exceptional in Morocco. Type 1 is the most common type. We noted through this study some diagnostic difficulties as the diagnosis was delayed and the enzymatic dosage was performed in only 42% of the cases as well as therapeutic difficulty with no prescription of the specific treatment given the high cost of the enzyme.

## Introduction

Gaucher's disease (GD) is an autosomal recessive genetic disorder that results from pathogenic mutations of the GBA gene encoding the enzyme glucocerebrosidase (acid β-glucosidase), which is located on chromosome 1q21.31. The absence or low activity of this enzyme leads to a progressive accumulation of its substrate (glucocerebroside) and hence causes the clinical manifestations of the disease [1]. GD is one of the most common lysosomal storage diseases and one of the rare genetic diseases for which therapy is now available. The purpose of this study is to define the epidemiological and clinical features of the disease and to highlight the diagnostic difficulties.

## Methods

We performed an 11-year (2002-2013) retrospective study of 11 patients with GD followed-up in the department of paediatric gastroenterology and nutrition of Rabat children's Hospital. The following baseline demographic characteristics were recorded for each patient in the study: age, sex, ethnicity, parental consanguinity, as well as the clinical, analytical, therapeutic and follow-up data.

## Results

We observed 11 patients with GD, 6 males and 5 females. The age at onset ranged from 3 months to 10 years with an average of 4 years. Parental consanguinity was noted in 85% cases. According to the clinical presentation, we classified our patients into: 9 cases of type 1 (81%) and two cases of type 2 (19%), none of the patients presented type 3.

### Patients with GD type 1

**Figure 1:**
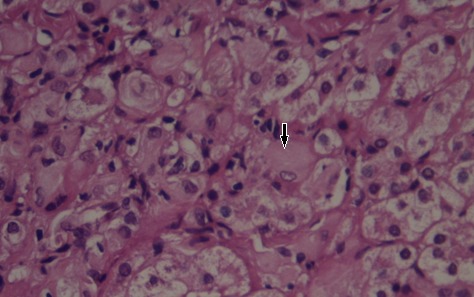
Liver (H&E stain × 40). Note parenchyma infiltration with Gaucher cells (arrow): large histiocytes with eccentrically placed nuclei

**Figure 2:**
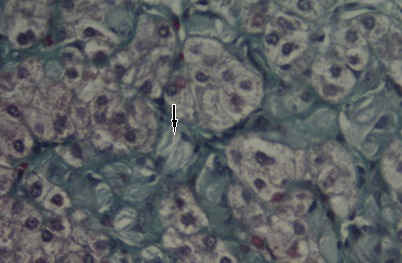
Liver (Special staining). Gaucher cell (arrow) with eccentrically placed nuclei and “crinkled paper” cytoplasm

**Figure 3:**
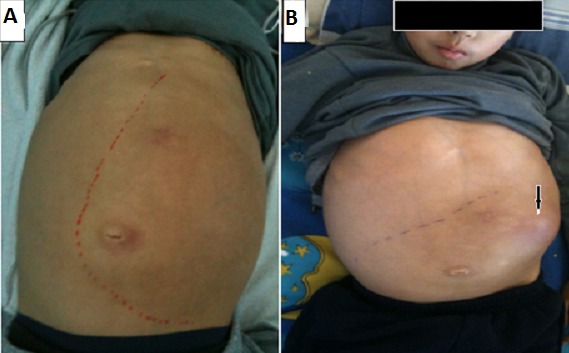
Patient at age 12. He presented with hepatosplenomegaly (A) and pancytopenia. Follow-up showed organomegaly worsening (B) with multiple splenic abscesses extended to the anterior abdominal wall (arrow)

**Figure 4:**
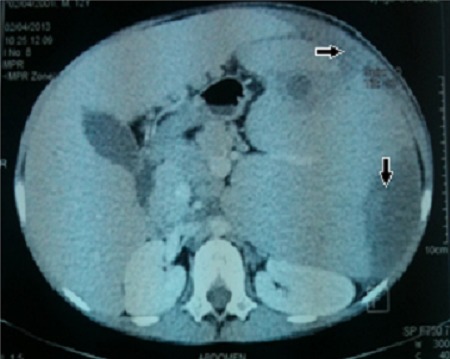
Abdominal CT scan showing the liver and spleen enlargement with splenic infarcts and abscess extended to the anterior abdominal wall (arrows)

**Figure 5:**
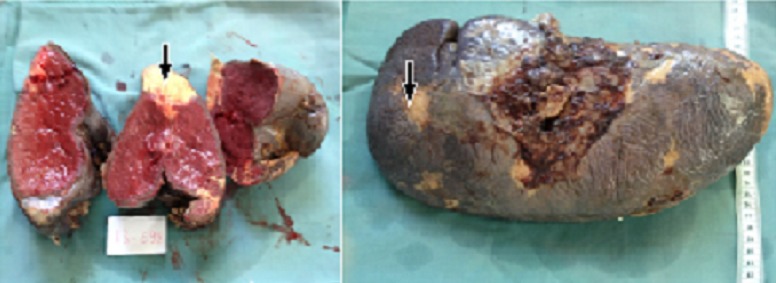
Spleen specimen. Note enlarged spleen with multiple infracts (arrows)

**Figure 6:**
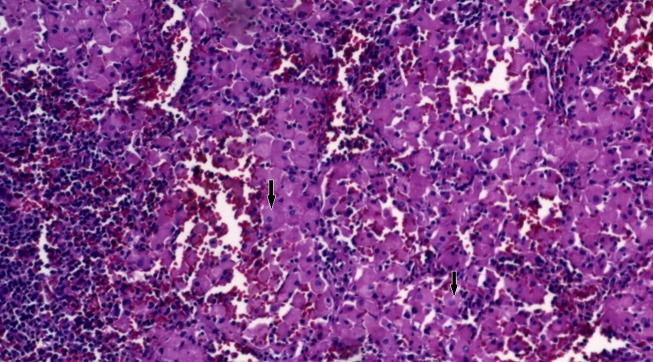
Cell analysis of splenectomy specimen. Note parenchyma infiltration with Gaucher cells (arrow)

We identified nine cases, 5 males and 4 females, the age at onset ranged from 2 to 10 years with an average of 5.2 years. The clinical manifestations are summarized in Table 1; splenomegaly and hepatomegaly were the main clinical symptoms. The hemogramm study revealed pancytopenia in 100% of cases: anaemia with hemoglobin < 100.000/mm^3^ in 56% of cases, leucopoenia with leukocyte count < 4000/mm^3^ in 56%. The diagnosis was based on histology showing the Gaucher's cells in various tissues, medullogram was performed in all cases and was positive in two cases; osteomedullary biopsy was conducted in four cases and was positive in three cases. All patients underwent a liver biopsy which was positive in all cases. (Table 2, Figure 1, Figure 2). Enzymatic activity dosage (glucocerebrosidase activity) was performed in 44.5% of cases. The treatment was based on symptomatic measures such as analgesics, transfusion and management of portal hypertension and its complications. Splenectomy was performed in one case presenting with multiple splenic abscesses and high transfusion requirements (Figure 3, Figure 4, Figure 5, Figure 6). None of the patients received a specific treatment (substitutive enzymotherapy). The follow-up period ranged from 3 months to 6 years with an average of 4 years. We noticed stability in 4 cases, two worsening cases with spleen and bone complications. Three patients were lost to follow-up.

**Table 1:** Main clinical features described in patients with GD type 1

Symptoms		Number	Percentage %
**Splenomegaly**		9	100
**Hepatomegaly**		9	100
**Fatigue**		7	78
**Haematological features**	Pallor	9	100
	Bleeding	4	44.5
**Bone manifestations**	Bone pain	3	33.5
	Fractures	2	22.5
**Failure to thrive**		6	67

**Table 2:** Diagnostic methods in GD type 1

	Number	Percentage (%)
Medullogram	2	22
Osteomedullary biopsy	3	34
Liver biopsy	9	100
Enzymatic dosage	4	44.5

### Patients with GD type 2

We observed two cases of GD type 2 diagnosed at 3 and 18 months. The visceral symptoms included hepatosplenomegaly, cytopenia and growth delay, neurological features comprised seizures in the first case and developmental milestones delay associated with hypertony and squint for the second case. Diagnosis was based on histopathological examination that highlighted Gaucher's cells in liver tissue. Treatment was purely supportive, both of the patients died, one month after diagnosis for the first case, and three months after diagnosis for the second case.

## Discussion

GD is a pan-ethnic disease and its worldwide prevalence is 1 in 50,000-100,000; however, it can be as high as 1 in 850 in individuals of Ashkenazi heritage [2]. In Morocco no survey has been conducted in order to accurately evaluate the prevalence, however, in our study high prevalence was noted in the north of the country (55%). The medical literature classifies GD into three broad categories: Type I, also referred to as adult or non-neuronopathic GD, which is characterised by the absence of significant neurological impairment; Type II or acute neuronopathic form, has very early clinical manifestations and significant neurological impairment; Type III or sub-acute neuronopathic form, is similar to type II, but less severe. This classification has been re-evaluated; some type 1 reported cases presented with neurological manifestation such as Parkinsonism [3], dementia and neuropathy [4]. Type 1 GD is by far the most common; it is estimated that less than 5% of patients with GD has type II or III GD [5]. In our study, classification was based on clinical presentation, although it showed predominance of type 1 GD (81%), future neurological involvement cannot be ruled out in some younger patients.

All 3 types of GD are inherited as autosomal recessive traits and have an equal sex distribution. Age at onset widely varies (from 1month [6] to 80 years [7]); some patients with type 1 GD may present early in childhood with virtually all the complications of GD, whereas others remain asymptomatic into the eighth decade of life after an incidental finding of thrombocytopenia or splenomegaly. Many affected individuals never develop signs or symptoms and do not seek medical attention. However, 66% of patients with type 1 GD are diagnosed before the age of 20 [8]. Types 2 and 3 GD typically present in early childhood. Some subjects with Parkinsonism have been found to have GD at a later age. In our study, mean age at onset was 3.41 year. Lag time between disease onset and diagnosis was determined to range from 1 month to 7 years, with an average of 2.3 years. The most important factor in the delayed diagnosis is lack of suspicion for the GD itself, this is related to disease rarity and variability of clinical presentation.

### Clinical manifestations

The clinical variability found in patients with GD is related to the type of mutation in the GBA gene and to the proteins, substrates and metabolism of each individual, as well as environmental factors; yet, many of these factors are not completely known. [9].

***GD type 1:*** at onset, patients with type 1 GD commonly present with highly variable degrees of splenomegaly, with the splenic tip extending to the pelvis. Enlargement of the spleen appears to be most rapid in children with Gaucher disease. Rapid enlargement of the spleen in an adult with the disease should prompt suspicion of an associated disorder that may increase glycolipid turnover, such as hematologic malignancy, immune thrombocytopenia, or autoimmune hemolytic anemia. Splenomegaly complications [10, 11] include hypersplenism with increased risk of infections, pain, rupture and infracts when the enlargement exceed 20-fold. In our study splenomegaly was constantly observed and complicated in one case with infracts ans abcesses (Figure 3, Figure 4). Hepatomegaly occurs in more than 50% of patients with type 1 GD [12, 13]. It was present in 100% of our cases. Minor elevations of liver enzyme levels are common, even in patients who are mildly affected, but the presence of jaundice or impaired hepatocellular synthetic function is a poor prognostic indicator. Jaundice in a patient with GD is usually a result of infection, the development of chronic hepatitis, or, rarely, hepatic decompensation in the late stages. Skeletal manifestations of GD vary, ranging from asymptomatic Erlenmeyer flask deformity of the distal femora to pathologic fractures, vertebral collapse, and acute bone crises that can be confused with acute osteomyelitis. Painful bone crises result from episodes of bone infarction, leading to osteosclerosis analogous to that occurring in sickle cell disease. In children with Gaucher disease, acute hip lesions can be misinterpreted as Legg-Calvé-Perthes disease, and avascular necrosis of the hips is a common complication in individuals of all ages. In our study, bone pain was present in 33.5% of cases with pathologic fractures in two cases.

Hematologic manifestations of GD include pancytopenia with anemia, thrombocytopenia and less commonly leucopenia [14]. Pancytopenia was present in all of our cases with secondary bleeding in 33.5% of cases. Numerous immunologic abnormalities are common in individuals with GD, including hypergammaglobulinemia, T-lymphocyte deficiency in the spleen, and impaired neutrophil chemotaxis. Other manifestations are noted such as the chronic fatigue and the short stature which is related to the energy expenditure required by the enlarged organs. The growth delay was observed in 67% of our patients. In addition unusual manifestations of type 1 GD are recognized such as increased risk of cancers, Parkinsonism and pulmonary hypertension [2].

***GD type 2:*** Type 2 disease is rare and is characterized by a rapid neurodegenerative course with extensive visceral involvement and death within the first 2 years of life. Patients with type 2 GD may present at birth or during infancy with increased tone [15], seizures [16], strabismus, swallowing abnormalities [17, 18], failure to thrive, and oculomotor apraxia; stridor due to laryngospasm are typical in infants with type 2 disease [19, 20]. The progressive psychomotor degeneration and brain stem involvement leads to death, usually caused by aspiration and respiratory compromise. A severe neonatal form can present in utero or perinatally with hydrops fetalis, congenital ichthyosis, or both. In our study, we observed two cases of type 2 GD. Beside the extensive visceral involvement; neurological features comprised seizures, developmental milestones delay, hypertony and strabismus.

***GD type 3:*** this form of GD widely varies and can present in infancy or childhood. Besides organomegaly and bony involvement, individuals with type 3 disease have neurologic involvement. The slowing of the horizontal saccades, an oculomotor finding, is often the sole neurologic manifestation. Some patients develop myoclonic epilepsy, exhibit learning disabilities, or develop dementia. None of our patients was diagnosed as type 3 GD.

### Laboratory Studies

***Enzyme activity testing*** [21]: diagnosis can be confirmed through measurement of glucocerebrosidase activity in peripheral blood leukocytes. A finding of less than 15% of mean normal activity is diagnostic. Heterozygotes generally have half-normal enzyme activity, but as much as 20% overlap with activity levels of healthy controls has been reported, rendering enzymatic testing for carrier status unreliable.

***Histologic Findings:*** The pathologic hallmark of GD is the presence of Gaucher cells in the macrophage-monocyte system, particularly in the bone marrow or in liver biopsy samples [21]. These cells have a characteristic wrinkled-paper appearance (Figure 1), resulting from intracytoplasmic substrate deposition, and stain strongly positive with periodic acid-Schiff. Histologic evaluation of biopsy specimens should not be used as a first-line diagnostic tool because the blood enzyme test is sensitive, specific, and much less invasive. However, enzymatic dosage in our study wasn′t always available, it was performed in only 36% of our cases (44.5% of type 1 GD cases), and therefore diagnosis was based on histological findings especially the liver biopsy (100% of cases).

***Genotype testing and phenotype-genotype correlation and complexity*** [21]: more than 200 different mutant GBA alleles have been identified in patients with GD [22, 23], the most frequent pathogenic mutations in the GBA gene are p.N370S (c.1226A > G) and p.L444P (c.1448T > C) [24]. Molecular diagnosis can be helpful, especially in Ashkenazi patients, in whom 6 GBA mutations (i.e., N370S, c.84insG, L444P, IVS2 + 1g > a, V394L, and R496H) account for most disease alleles. In other ethnicities, sequencing of the exons of GBA is necessary in order to accurately establish the genotype. Mutation analysis has some, albeit limited, predictive value with respect to disease progression. For example, patients with type 1 GD who are homozygous for the N370S mutation tend to have a later onset and a relatively mild course, and patients with type 3 GD who are homozygous for the D409H mutation exhibit a rare phenotype that involves cardiac calcifications, oculomotor abnormalities, and corneal opacities. However, clinical presentation in patients with GD widely varies and frequently cannot be fully explained by the underlying mutations because severity can vary even among siblings who have identical genotypes. Despite recognizing its high importance, genetic testing wasn′t performed in our study because of its unavailability in Morocco and hence its high cost.

### Management

For many years, GD was treated with symptomatic therapies and palliative measures such as transfusion, analgesics and splenectomy. Enzyme replacement therapy (ERT) is now available and includes imiglucerase (Cerezyme), velaglucerase alfa (VPRIV), and taliglucerase alfa (Elelyso). Most patients receive the recombinant enzyme imiglucerase [25]. The response to this preparation differs according to: a) type of GD (type I or III); b) initial degree of involvement; and c) affected organs. In general, the best response is obtained in the hematological and visceral parameters. Pulmonary involvement and some aspects of the bone disease, such as osteonecrosis, osteofibrosis, and lytic lesions, cannot be reversed. Nevertheless, early initiation of treatment reduces the risk of these irreversible complications [2]. Gene therapy may be a future step. No evidence shows that ERT results in neurologic improvement. Although the enzyme affects the visceral involvement in types 2 and 3 disease, the associated brain involvement may persist or progress. ERT is highly effective in improving the quality of life [26], growth velocity, weight gain and energy levels; other effects include a correction of both delayed puberty and hypermetabolic state. Oral substrate reduction therapy, using an aminosugar inhibitor of glucosylceramide synthase, has been approved for use in patients with type 1 GD in whom ERT is not a therapeutic option because of allergy, hypersensitivity, or poor venous access. Although oral substrate reduction therapy was approved, long-term data regarding efficacy and safety are not yet available. Despite all the benefits of the ERT, our patient′s access to this therapy remained impossible due to its high cost, in fact, for a patient whose weight is 66 pounds the annual cost may reach 300.000 dollars. Therefore, the treatment in our study was always symptomatic (analgesics, transfusion). Splenectomy was performed in one case presenting with multiple splenic abscesses and high transfusion requirements.

### Prognosis

A recent study estimated life expectancy at birth in patients with type 1 GD to be 68 years, compared with 77 years in the reference population [27]. The prognosis for symptomatic patients with type 1 or type 3 GD who receive treatment is very good, with a decrease in organomegaly and an eventual rise in hemoglobin levels and platelet counts. The prognosis in type 2 GD is severe; although ERT affects the visceral involvement, brain involvement is fatal and may persist or progress. In our study; the follow-up period of patients with type 1 GD ranged from 3 months to 6 years with an average follow-up of 4 years. We noticed stability in 4 cases, 2 worsening cases with bone and spleen complications, and 3 patients were lost to follow-up. On the other side; both of type 2 GD cases died respectively one month and three months after diagnosis.

## Conclusion

Gaucher's disease is not exceptional in Morocco. Type 1 is the most common type. We noted through this study diagnostic delay and insufficiency as the enzymatic dosage was performed in only 36% of the cases as well as therapeutic difficulty with no prescription of the specific treatment given the high cost of the enzyme.
